# TranSpec3D: A Novel Measurement Principle to Generate A Non-Synthetic Data Set of Transparent and Specular Surfaces without Object Preparation

**DOI:** 10.3390/s23208567

**Published:** 2023-10-18

**Authors:** Christina Junger, Henri Speck, Martin Landmann, Kevin Srokos, Gunther Notni

**Affiliations:** 1 Group for Quality Assurance and Industrial Image Processing, Technische Universität Ilmenau, 98693 Ilmenau, Germany; 2 Fraunhofer Institute for Applied Optics and Precision Engineering IOF Jena, 07745 Jena, Germany

**Keywords:** deep stereo matching, monocular depth estimation, data set, real, non-synthetic, object preparation, object painting, non-Lambertian surface, transparent, specular, thermal 3D sensor, TMRP

## Abstract

Estimating depth from images is a common technique in 3D perception. However, dealing with non-Lambertian materials, e.g., transparent or specular, is still nowadays an open challenge. However, to overcome this challenge with deep stereo matching networks or monocular depth estimation, data sets with non-Lambertian objects are mandatory. Currently, only few real-world data sets are available. This is due to the high effort and time-consuming process of generating these data sets with ground truth. Currently, transparent objects must be prepared, e.g., painted or powdered, or an opaque twin of the non-Lambertian object is needed. This makes data acquisition very time consuming and elaborate. We present a new measurement principle for how to generate a real data set of transparent and specular surfaces without object preparation techniques, which greatly reduces the effort and time required for data collection. For this purpose, we use a thermal 3D sensor as a reference system, which allows the 3D detection of transparent and reflective surfaces without object preparation. In addition, we publish the first-ever real stereo data set, called TranSpec3D, where ground truth disparities without object preparation were generated using this measurement principle. The data set contains 110 objects and consists of 148 scenes, each taken in different lighting environments, which increases the size of the data set and creates different reflections on the surface. We also show the advantages and disadvantages of our measurement principle and data set compared to the Booster data set (generated with object preparation), as well as the current limitations of our novel method.

## 1. Introduction

Transparent and specular objects are omnipresent and belong to optically uncooperative objects in the visual spectral range (VIS). Representatives are various glass objects, e.g., glass walls or glass flasks, and transparent or translucent plastic parts, e.g., clear orthodontic aligners or car headlights. Typical areas of application are as follows: (a) human–robot interactions, e.g., for confidential detection of visually uncooperative objects [1]; (b) autonomous robot navigation, e.g., collision prevention of glass walls; (c) laboratory automation, e.g., for grasping visually uncooperative objects [2,3,4,5]; (d) medical section, e.g., 3D reconstruction of clear orthodontic aligners; (e) autonomous waste sorting and recycling, and (f) augmented reality [6]. In these use cases, there are two main tasks:

- Locating optically uncooperative objects. This includes object segmentation [7,8] and object pose estimation [9,10,11].
- Accurately estimating the depth of optically uncooperative objects. This includes accurate and reliable depth estimates, also known as deep depth completion [2,12,13,14], 3D reconstruction methods [3,15,16], and stereo vision [17,18,19].

This paper describes the current challenges in the stereo depth estimation of transparent and specular objects and presents a new measurement principle for the acquisition of real ground truth data sets.

**Figure 1:**
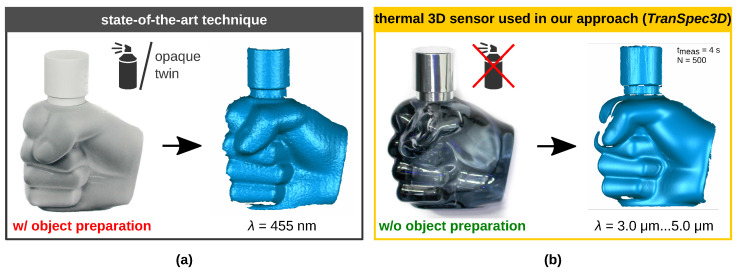
Two techniques to three-dimensionally record transparent, translucent, and reflective surfaces [31]. Object: fist-shaped glass flacon with metal-covered plastic cap. (**a**) State-of-the-art technique: Using an active VIS 3D sensor requiring object preparation (diffuse reflective coating) [11,17,18,27,28]. (**b**) Alternative measurement technology: Using a thermal 3D sensor [30] without object preparation. Wavelength of stereo system λ; measuring time $t_{\mathrm{meas}}$; number of fringes *N* (sequential fringe projection).

The conventional 3D sensors in the VIS and near-infrared (NIR) spectral range are not suitable for the perception of transparent, translucent, and reflective surfaces [2,6,13,17,19,20] since stereo matching, i.e., the search for correspondence points in the left and right image, is error prone [18,19]. The limitations are described in detail in Section 2.1. To overcome this limitation, data-driven approaches of artificial intelligence (AI)-based stereo matching methods [17,18,21] or monocular depth estimation [22,23,24] are applied. In the process, known (uncooperative) objects that were set during the training time can be perceived without object preparation (also called in distribution). However, there are currently two challenges (A) and (B) for deep stereo methods for visually uncooperative surfaces.

(A) This method requires a large training and test data set with ground truth disparity maps. Synthetic data sets or real data sets can be used. Real data sets, unlike synthetic data sets [2], capture the environment most realistically but are difficult [2], very time consuming and expensive to create [18,25]. That is why hardly any real ground truth data sets exist. The most complex part is the generation of the ground truth, so-called annotation. Therefore, optically uncooperative objects are prepared (e.g., diffuse reflective coating) in order to optically detect them in the VIS or NIR spectral range [17,18,26,27,28]. Figure 1a shows that the manipulated surface can thereby be captured three-dimensionally. **This technique is very elaborate and very highly time consuming due to the object preparation process**  [18,25]; see Figure 1a. This process also includes **high effort in positioning prepared objects to the previous place of unprepared objects** [25]**, and possible object cleaning**. **Object preparation is not suitable or appropriate for many objects that may not be prepared**, such as historical glass objects.
(B) The transparency awareness ability is a corner case in deep stereo matching networks [17,19]. Furthermore, current deep stereo matching approaches—regardless of the challenge with transparent objects—are generally limited to a specific data set due to divergent key factors in multiple data sets, and generalize poorly to others. Three key factors are unbalanced disparity distributions and divergent textures and illuminations [20,29].

The performance of deep stereo matching networks is strongly dependent on the performance of the training data [29]. (The definition and key role of the performance of the data set are described in detail in Section 2.2.) For deep stereo matching and monocular depth estimation, data sets with ground truth are needed. Synthetic data can be produced cheaply without complex surface preparation in large quantities compared to real data sets. Therefore, more synthetic data sets are available than real ones [6]. In order to synthesize a representative data set of transparent objects and artifacts such as specular highlights and caustics, however, very high-quality rendering and 3D models are required [2]. Real data sets are preferable to synthetic data sets for the following reasons:

- Real data are authentic and reflects the real world;
- Real data contain errors, inaccuracies and inconsistencies;
- Real data represent human behavior or complex interactions better.

Nevertheless, real-world ground truth data sets for optically uncooperative objects are difficult to obtain [2], still time consuming and expensive [18,25] due to the necessary object preparation (see Section 2.1). Yet, there are hardly any available real-world data sets for stereo systems suitable for disparity estimation (Table 1) as well as for mono depth estimation (Table 2). Table 1 shows an overview of real-world (non-synthetic) stereo data sets with transparent and specular objects **suitable for disparity estimation**. Liu et al. [25] created the *Transparent Object Data Set* (TOD) for pose estimation and depth estimation. The generation of the ground truth data set is very time consuming and elaborate. The ground truth depth of the transparent object was acquired in an additional step with an opaque twin in the same position as the previously acquired transparent object. The challenging part is the exact placement of the opaque twin at exactly the same position as the transparent object. Ramirez et al. [18] created the first real stereo data set for transparent and reflective surfaces, named *Booster*. The ground truth disparity map is obtained by additionally preparing the scene. All non-Lambertian surfaces in the scene are painted or sprayed to allow the projection of textures over them. Our *TranSpec3D* data set is, according to our research, the first real (stereo) data set for visually uncooperative materials generated without object preparation, e.g., white titanium dioxide powder. **This shortens and simplifies the ground truth data acquisition process by eliminating the need for object preparation and the accurate placement of the respectively prepared opaque twin object** (cf. [18,25]). With our novel measuring principle, we overcome the challenges (A). For this purpose, we additionally use a thermal 3D sensor developed by Landmann et al. [30] to generate real ground truth data. With this additional sensor, the fast and easy acquisition of real ground truth data is possible with little effort and without object preparation; see Figure 1b.

Figure 1b shows an alternative method published by Landmann et al. [30] for measuring freeform surfaces without any object preparation with high accuracy. The object surface is heated up locally by only a few Kelvin under the generation of a heat pattern. The surface itself emits this heat pattern which is recorded by thermal cameras. Like in VIS or NIR, the camera pictures are evaluated, and a 3D shape is reconstructed. The fully automatic 3D reconstruction takes place within seconds [30]. Three disadvantages of this technology, however, are the high hardware costs, the necessary safety-related enclosure and the longer measurement time compared to conventional stereo systems. Objects with very high thermal conductivity or good thermal conductors are not measurable. Nevertheless, with higher costs, the measurement time can also be reduced. There is still potential for development to remedy some disadvantages.

The main contributions of our work are as follows:

- We introduce a novel measurement principle *TranSpec3D* to generate for transparent and specular objects the **first-ever** real data set with ground truth without object preparation (e.g., object painting or powdering). **The absence of object preparation greatly simplifies the creation of the data set, both in terms of object reusability and time, as there is no need to prepare the objects or generate opaque twins, including drying and accurately placing the non-prepared and prepared objects** (cf. [2,12,18,25]). In addition, the surface of the object is not manipulated (cf. [18,25]). For data set generation, any conventional 3D sensor is supplemented by a thermal 3D sensor developed by Landmann et al. [30]. The thermal 3D sensor captures the optically uncooperative objects three-dimensionally without time-consuming object preparation. This measurement principle can be used to generate real monocular as well as stereo data sets, which can be applied, e.g., to monocular depth estimation (depth-from-mono) [4,23,24] or deep stereo matching [21].
- Based on the new measurement principle, we created a new real-world (non-synthetic) stereo data set with ground truth disparity maps, named *TranSpec3D*. Our data set is available at https://QBV-tu-ilmenau.github.io/TranSpec3D-web (accessed on 6 September 2023).

## 2. Background Information

### 2.1. Limitation of Three-Dimensional Perception of Transparent Objects

**Figure 2:**
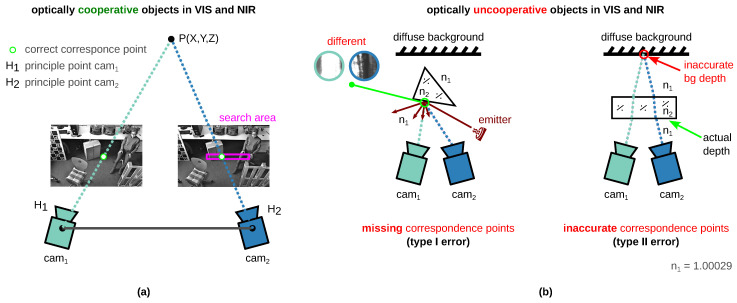
(**a**) Searching algorithm of the correspondence of optically cooperative features in VIS and NIR (Lambertian surface). Spanning triangle $\Delta \;H_{1}H_{2}P$ with the two principle points $H_{1/2}$ and the actual 3D point P(X,Y,Z) (according to [33]). (**b**) Limitations in stereo matching due to optically uncooperative features in VIS and NIR (non-Lambertian). Two typical depth errors are shown (cf. errors in RGB-D sensors, according to [2,6]). Type I error (missing depth): typically occur due to (1) specular reflections on the uncooperative surface or due to (2) the detection of different background areas behind the transparent surface. Type II error (inaccurate depth): due to the detection of the same point in the background (bg) area behind the transparent surface. Instead of the actual surface, the background is captured. Incidentally, the background has an inaccurate depth due to the change in the refractive index, and therewith in the direction of the intersecting rays. n1$\hat{=}$ refractive index of air; n_2_$\hat{=}$ refractive index of optically uncooperative surface, e.g., glass.

The conventional 3D sensors in the VIS and near-infrared (NIR) spectral range are not suitable for the perception of transparent, translucent, and reflective surfaces [2,6,13,17,19,20] since stereo matching, i.e., the search for correspondence points in the left and right image, is error prone [18,19]. Figure 2 shows the comparison of stereo matching with optically cooperative (a) and uncooperative (b) objects in VIS and NIR. Two errors can occur when detecting uncooperative objects (non-Lambertian surface). In the case of *depth error I* (missing depth), for example, no depth values can be determined due to specular highlights on the surface [2,6,18]. In case of *depth errors II* (inaccurate/background depth), the same point (or points with the same feature) of the background surface behind the transparent object is detected instead of the actual optically uncooperative object surface. For our problem, it is irrelevant that the measured depth value is also inaccurate. Error II is unfavorably named “background error” by Jiang et al. [6] (RGB-D sensor). In the case of a stereo system, this can lead to misunderstanding (see following text). It is therefore better to name it as an “inaccurate error” as used in [2] for RGB-D sensors.

**Figure 3:**
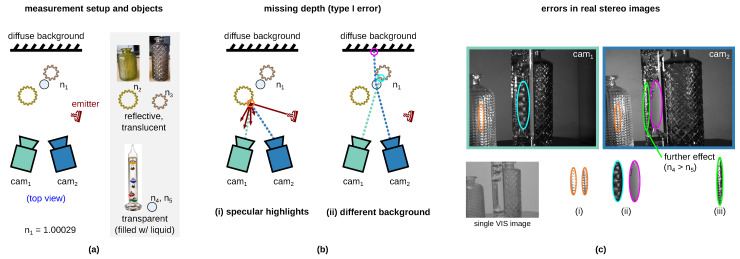
Type I error (missing depth) in conventional stereo matching [21,34] (VIS or NIR) of visually uncooperative objects. (**a**) Measurement setup consisting of a passive NIR 3D sensor (convergent camera setup) and two NIR emitters to produce different reflections. Objects: two reflective and translucent vases and transparent glass Galileo thermometer (filled with liquid). n_1_ $\hat{=}$ refractive index of air. (**b**) missing depth error “occure due to (i) specular reflections on the transparent surface” [6] or due to (ii) the detection of different background areas behind the transparent surface. The background is distorted by anything that has a different refractive index than air n_1_ and an optical thickness. (**c**) Rectified stereo image (NIR) of *TranSpec3D* data set with drawn-in errors (i) and (ii) and further effect (iii). Further effect: Total reflection at the interface 4-5, due to refractive index difference between glass n_4_ and liquid n_5_ (n_4_ > n_5_).

**Figure A1:**
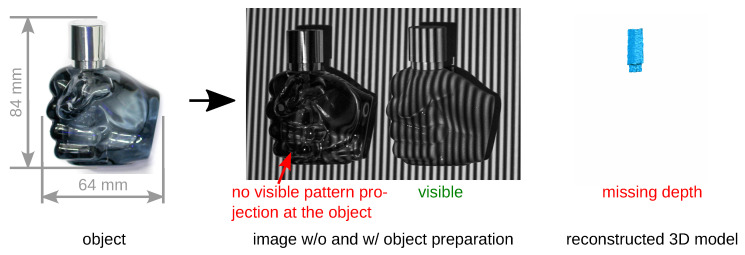
Limitations of active 3D sensors in VIS and NIR with optically uncooperative objects. Missing depth due to non-visible pattern projection in VIS or NIR (according to [31]). Object: fist-shaped glass flacons with metal-covered plastic cap.

The type I error occurs very frequently and can occur due to different effects. Figure 2 shows different depth errors due to missing depth on the example of our rectified stereo images from our novel *TranSpec3D* data set (passive NIR 3D sensor). Figure 2a shows the measurement setup consisting of our (passive) NIR 3D sensor and two NIR emitters. The scene contains two reflective, translucent vases and a transparent Galileo thermometer filled with a liquid. Figure 2b shows missing depth errors due to (i) specular highlights on the surface or (ii) the detection of different background areas behind the transparent object. The background is distorted by transparent objects that have different refractive indices than surrounding air $n_{1}$. Case (ii) is not to be confused with the “inaccurate/background error” (type II), although here, two different backgrounds (vase and diffuse background) are optically detected. In addition, further effects can occur, (iii) such as total reflection at the interface 4–5, due to the refractive index difference between glass n_4_ and liquid n_5_ (n_4_ > n_5_; see Figure 3c). Furthermore, (iv) with active VIS or NIR 3D sensors, the projection pattern onto the surface of the transparent object is not visible (see Figure A1, Appendix A) [31].

### 2.2. Key Role of Data Set

Data sets play a key role in AI-based image-based approaches as well as for the required hardware. This is because the performance of deep learning networks (data driven) is directly dependent on the performance of the data set. The performance is defined by the scale, quality and speed. *Scale* stands for the resolution and dimension of the images as well as the size of the data set. As the image dimensions increase, the disparity range and the number of occluded and unstructured pixels also increase, making it difficult to find the accurate corresponding pixels. In addition, high-resolution images are a limiting factor for most stereo matching as well as monocular state-of-the-art networks. Figure S1 shows the strange effects of resolution. The processing is much more complex compared to low-resolution images and requires networks that feature larger receptive fields and reason at multiple context levels [18,35]. The receptive field must contain contextual clues, e.g., margins, and occlusions [35]. Miangoleh et al. [35] developed a new approach for *monocular depth estimation* to solve this problem. *Quality* stands for data without any defects, such as blur, noise, distortion, or misalignment, the selection of meaningful modalities and the consistent and accurate ground truth (annotation). For ground truth stereo data, this means without missing and inaccurate correspondence points (cf. Figure 2). The relevance of real data sets is described in Section 1. In addition, a reasonable minimum selection of meaningful modalities reduces the load on subsequent networks by having fewer dimensions and supports the networks by having better quality features. *Speed* stands for the generation time of data collection. When creating real data sets for transparent and specular objects, this time can be reduced with our novel *TranSpec3D* measurement principle (Section 3), as object preparation is no longer necessary. Due to the reducing time requirements, the size as well as the number of freely available real data sets for optically uncooperative objects will presumably increase in the future.

## 3. TranSpec3D Measurement Principle

In this section, we report in detail our novel measurement principle and experimental setup (Section 3.1), as well as the method for generating the data set (Section 3.2).

### 3.1. Measurement Principle and Experimental Setup

Our goal is to create a real data set (for monocular or stereo systems) with ground truth depth or disparity maps for transparent, translucent and specular objects without the time-consuming and cost-intensive state-of-the-art object painting [18]. We achieve this by extending our conventional stereo system with an alternative sensor technology (thermal 3D sensor [30]) that can detect these objects, which are optically uncooperative in the VIS, in three dimensions. The conventional stereo system can be replaced by a monocular camera or another 3D sensor, e.g., an RGB-D sensor.

**Figure 4:**
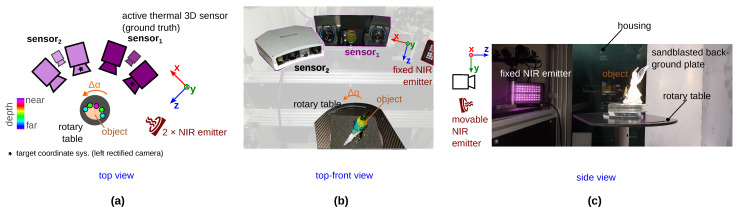
Measurement setup of *TranSpec3D* data set. (**a**) Top view of setup, consisting of a thermal 3D sensor (sensor_1_) and an NIR 3D sensor (sensor_2_), two NIR emitters and a rotary table so that the two 3D sensors can measure the object from the same viewing direction. The rotary table is turned by Δα. (Any 3D sensor, e.g., active/passive stereo system or RGB-D sensor, can be utilized as sensor_2_.) (**b,c**) Top–front view and side view of our setup with a measurement volume of 160 mm×128 mm×100 mm. Our background [37] is diffusely reflective with an angle of 30${}^{\circ }$ (**c**).

Figure 4 shows our experimental setup, consisting of the thermal 3D sensor (sensor_1_, $\lambda_{{\mathrm{sensor}}_{1}}=3-5\;\mu m$) for recording the actual measurable ground truth depth and a conventional stereo system sensor_2_. The objects are detected in this technology (sensor_1_) by re-emission. The result is a low resolution point cloud of about 0.3 Mpx (see Section 3.2.4). As sensor_2_, we use a conventional active NIR 3D sensor based on GOBO (GOes Before Optics) projection ($\lambda_{{\mathrm{sensor}}_{2}}=\;850\;nm$) [36], whereby we only use the projection to set up the system (see Section 3.2.1) but not to generate the stereo images (with 1.9 Mpx image resolution). Furthermore, we use an additional monocular camera (VIS) camera_3_. With this camera, it is possible to create a monocular data set in the VIS spectral range. Table A2 (Appendix C) shows the properties of the 3D sensors. In order to have the same viewing angle of the static object from both 3D sensors, we arranged the sensors horizontally next to each other and integrated a rotary table. To avoid inaccuracies due to a backlash of the turning axis, only one direction is rotated (mathematical direction of rotation). Furthermore, we use two NIR emitters (Instar IN-903 at λ=850 nm) to achieve the variance in the stereo images (sensor_2_) through different specular reflections on the objects (natural data augmentation).

### 3.2. Generation of Data Set

**Figure 5:**
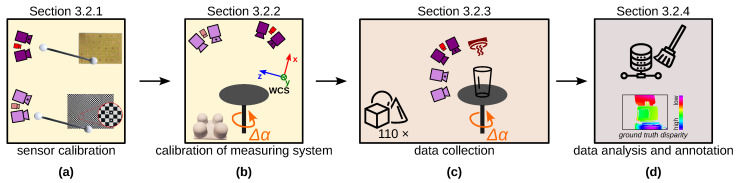
Our data capturing and annotation pipeline for *TranSpec3D* stereo data set: (**a**) sensor calibration of (top) thermal 3D sensor and (bottom) NIR 3D sensor; (**b**) calibration of NIR 3D sensor, thermal 3D sensor, and the axis of the rotary table (turned by Δα) in world coordinate system (wcs) using different test specimens, e.g., a 4-hemisphere specimen; (**c**) data collection of 110 different objects; (**d**) data analysis and annotation (generation of ground truth depth or disparity maps).

Figure 5 shows our data capturing and annotation pipeline: (a) sensor calibration; (b) estimate extrinsic parameters of measuring system; (c) data collection; (d) data analysis and annotation of ground truth disparity maps. Step (a) is described in Section 3.2.1, step (b) in Section 3.2.2, step (c) in Section 3.2.3 and step (d) in Section 3.2.4.

#### 3.2.1. Sensor Calibration

**Figure A2:**
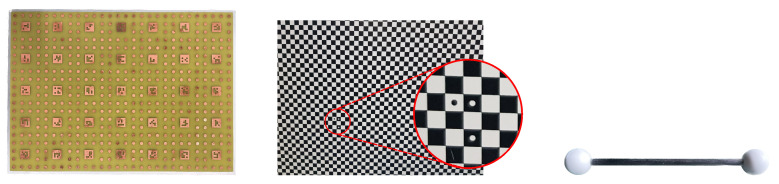
Planar calibration targets for *TranSpec3D* data set setup. (**left**) Calibration target (circuit board FR-4) with copper-coated ArUco markers and symmetrical circles for sensor_1_ (thermal 3D sensor [30]), (**mid**) checkerboard with “3-circle marking” for sensor_2_ (NIR 3D sensor) on glass plate. (**right**) Sphere bar, for determination of the skew factor of sensor_2_ (Ser. No. 2016; Ingenieria y Servicios Metrologia Tridimensional S.L.).

Each system is first calibrated separately with different planar calibration targets (see Figure A2, Appendix D). To increase the accuracy, sensor_2_ is used as an active stereo system, i.e., with GOBO projection. Table 3 shows the calibration details (method, target, etc.). The calibration *scrw-factor* is calculated using a spherical bar (Figure A2, right). This value is measured in one plane by default. Actually, however, this should be determined over the entire measuring field. There is no method for this yet. The rectification for sensor_2_ takes place via the openCV function stereoRectify(). After rectification, the reprojection matrix resp. disparity-to-depth mapping matrix $Q_{{\mathrm{sensor}}_{2}}$ for the sensor_2_ (NIR 3D sensor) is available. $Q_{{\mathrm{sensor}}_{2}}$ contains the focal length (f), the principal points of the left ($cx_{1},\;cy_{1}$) and right ($cx_{1},\;cy_{1}$) rectified cameras, and the horizontal base distance $T_{x}$ of the cameras. The components of $Q_{{\mathrm{sensor}}_{2}}$ are used to create the two 3×4 projections matrices $P_{\mathrm{rect}1/2}$; see Equation (1). These are mandatory for the conversion of the depth values into disparity values (Section 3.2.4). After the sensor calibration, the systems are ready for measurement. The depth values of the point clouds are in mm:

(1) $$\begin{array}{c} P_{\mathrm{rect}1}=\left(\begin{array}{cccc} f & 0 & cx_{1} & 0\\ 0 & f & cy_{1} & 0\\ 0 & 0 & 1 & 0 \end{array}\right),\;P_{\mathrm{rect}2}=\left(\begin{array}{cccc} f & 0 & cx_{2} & T_{x}\;\cdot \;f\\ 0 & f & cy_{1} & 0\\ 0 & 0 & 1 & 0 \end{array}\right) \end{array}$$

#### 3.2.2. Calibration of the Measuring System

**Figure 6:**
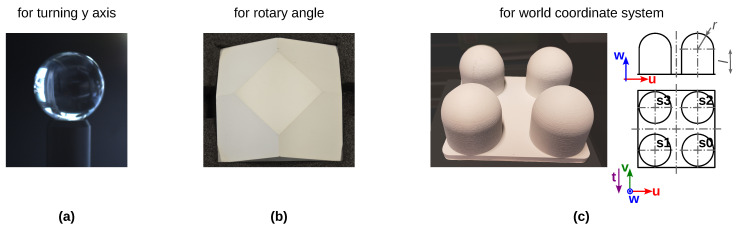
Utilized test specimens for calibration of *TranSpec3D* data set setup. (**a**) Glass ball for determining and calibrating the axis of rotation of the rotary table. (**b**) Prism with frontal surface in matte white for determining the angle of rotation Δα. (**c**) (**left**) Four-hemisphere for determination of transformations to the global world coordinate system (wcs). (Filament of 3D printing part: colorFabb PLA ECONOMY SILVER 2.85 mm; Painted with pure white matte RAL 9010). (**right**) Technical drawing; l=60.1 mm; r=40 mm; center of the hemispheres: s0–s3. The vectors u, v and w describe the rotation matrix $R_{\mathrm{wcs}}^{\mathrm{gt}}$.

In the following, details of the calibration of the axis of the rotary table, rotary angle Δα, thermal 3D sensor and NIR 3D sensor in the world coordinate system (wcs) are described. Figure 6 shows the test specimens utilized for this purpose. Test specimen (a), a glass sphere, is for the determination of the turning axis ($r_{\mathrm{axisY}}$ and $t_{\mathrm{cntrP}}$), (b) is for the determination of rotary axis Δα, and (c) (four hemispheres) is for the determination of the world coordinate system (wsc). Table A4 shows the set and actual values of the specimen (c). Below, we describe the determination of the remaining parameters of the *TranSpec3D* sensor homogeneous coordinate transformations.

##### Determinationof the Turning Axis of the Rotary Table

**Figure 7:**
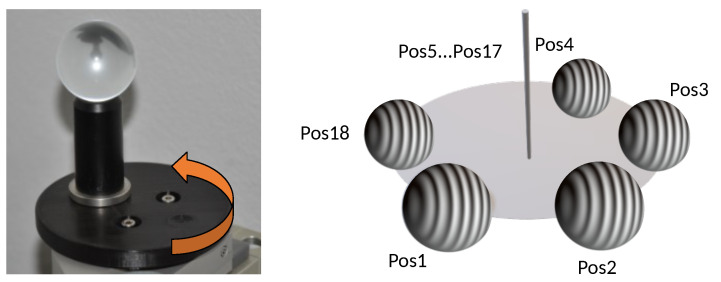
Principle for determining and calibrating the rotation axis of the rotary table. For our data set, we measured the test specimen (glass ball) at 18 different positions Pos1 to Pos18 (by turning the turntable). The rotary table shown on the left is different from the rotary table used in our *TranSpec3D* data set. However, the test specimen is the same.

The specimen (a) is placed on the rotary table (see Figure 6). Eighteen measurements are performed at different positions of the rotary table (see Figure 7). For each measurement position, the sphere center is determined in the 3D point cloud. A circle is then fitted from the sphere centers, where its center and the normal result in the center of rotation $t_{\mathrm{cntrP}}$ and the axis $r_{\mathrm{axisY}}$. Equation (2) describes the turning *y*-axis and the rotary angle Δα:

(2) $$r_{\mathrm{axisY}}={\left(\begin{array}{ccc} r_{x} & r_{y} & r_{z} \end{array}\right)}^{T},\;\;t_{\mathrm{cntrP}}={\left(\begin{array}{ccc} x_{\mathrm{cntrP}} & y_{\mathrm{cntrP}} & z_{\mathrm{cntrP}} \end{array}\right)}^{T},\;\;\Delta \alpha =31.4{}^{\circ }$$

##### Determination of the Rotary Angle of Rotary Table

To determine the rotary angle Δα, the specimen (b) is applied (see Figure 6). The flat frontal surface of the specimen is aligned orthogonally to the optical axis of the sensor_1_. The specimen is then detected in this position by both 3D sensors. After that, the same is performed, except that the flat frontal surface of the specimen is aligned orthogonally to sensor_2_. With the help of the recorded 3D points and the number of steps of the motor, we can infer the angle Δα.

##### Determination of Transformations to the World Coordinate System

For this purpose, a four hemisphere specimen is utilized; see Figure 6c. The test specimen is detectable for both 3D sensors. Homogeneous coordinate transformation (wcs-to-gt): Both calibrated 3D sensors synchronously capture the test specimen (c) by a rotary angle (rotary table) of $\alpha =0{}^{\circ }$. In the acquired point clouds, the centers of the four hemispheres of the specimen are determined (s0,s1,s2,s3), see Equation (3), by fitting a sphere per hemisphere and determining the center from it. We define the center of these spherical centers as our world coordinate system (wcs):

(3) $$s0=\left(\begin{array}{c} X_{s0}\\ Y_{s0}\\ Z_{s0} \end{array}\right),\;\;s1=\left(\begin{array}{c} X_{s1}\\ Y_{s1}\\ Z_{s1} \end{array}\right),\;\;s2=\left(\begin{array}{c} X_{s2}\\ Y_{s2}\\ Z_{s2} \end{array}\right),\;\;s3=\left(\begin{array}{c} X_{s3}\\ Y_{s3}\\ Z_{s3} \end{array}\right)$$

The translation vector $t_{\mathrm{wcs}}^{\mathrm{gt}}$, Equation (6), is the midpoint of the four spherical centers (s0,s1,s2,s3); see Equation (4). With the help of the spherical centers (s0,s1,s2), we can calculate vectors u, v and w; see Equation (5). These vectors describe the rotation matrix $R_{\mathrm{wcs}}^{\mathrm{gt}}$. See Equation (4):

(4) $$sx=\frac{\sum_{i=0}^{3}X_{si}}{4},\;\;sy=\frac{\sum_{i=0}^{3}Y_{si}}{4},\;\;sz=\frac{\sum_{i=0}^{3}Z_{si}}{4},\;\;\mathrm{with}\;i\in \left[0,1,2,3\right]$$

(5) $$t=\frac{s2-s0}{∥s2-s0∥},u=\frac{s1-s0}{∥s1-s0∥},w=[t\;✕\;u],v=[w\;✕\;u]$$

(6) $$R_{\mathrm{wcs}}^{\mathrm{gt}}=\left(\begin{array}{c} u\\ v\\ w \end{array}\right),\;\;t_{\mathrm{wcs}}^{\mathrm{gt}}=\left(\begin{array}{c} sx\\ sy\\ sz \end{array}\right)$$

Homogeneous coordinate transformation (wcs-to-S): The determination of the rotation matrix $R_{\mathrm{wcs}}^{S}$ and the translation vector $t_{\mathrm{wcs}}^{S}$ is analogous to the calculation of the homogeneous coordinate transformation (wcs-to-gt). The only difference is the different rotary angle $\alpha +=31.4{}^{\circ }$ of the rotary table at data collection.

#### 3.2.3. Data Collection

**Figure 8:**
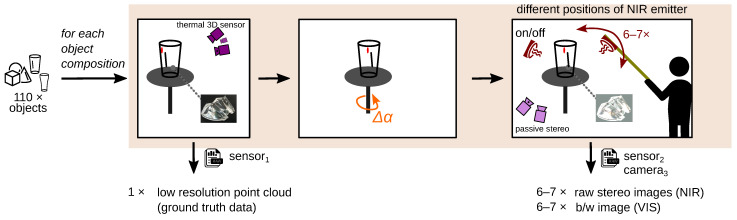
Data collection: Pipeline per object or object composition. (**left**) Data acquisition of the object with sensor_1_ thermal 3D sensor (ground truth depth); (**mid**) alignment of the object to the NIR sensor by rotary table by rotary angle Δα. (**right**) images taken with sensor_2_ (NIR 3D sensor w/o GOBO projection) at different illumination positions. This is for natural data augmentation.

Figure 8 shows the data collection process per object. After placing the objects on the rotary table, the scene is captured with the thermal 3D sensor (sensor_1_). The output is a point cloud, which is our ground truth depth. Then, the rotary table is rotated by $\Delta \alpha =31.4{}^{\circ }$. Then the data acquisition is performed with the NIR 3D sensor without GOBO projection (sensor_2_). On average, this scene is acquired with six to seven different positions of the two NIR emitters. In this way, we increase our data set and the variance regarding specular reflections. Therefore, there are several stereo images for this scene under different lighting conditions.

#### 3.2.4. Data Analysis and Annotation

**Figure 9:**
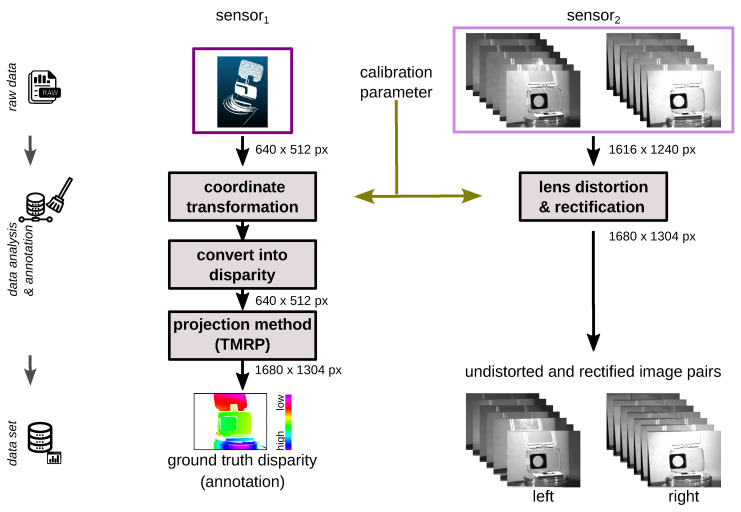
Data analysis and annotation: (**left**) Processing pipeline: transformation of the low resolution depth point cloud of sensor_1_ to the left rectified camera of sensor_2_, calculation of disparities and projection of points into 2D raster image using TMRP method [39]. (**right**) Pipeline of stereo images of sensor_2_. Object: (**mid**, **top-down**) Transparent waterproof case for action camera, Petri dish (glass) and polymethyl methacrylate (PMMA) discs with different radii.

Figure 9 describes the data analysis and annotation of the ground truth disparity maps. For the raw stereo data of sensor_2_, a lens distortion correction and rectification is performed. The calibration parameters (see Section 3.2.1 and Section 3.2.2) are used for this. The raw point cloud of sensor_1_ is first transformed into the coordinate system of sensor_2_ based on the calibration parameter. Then, the depth values are converted into disparity values. The resulting point cloud is projected into a 2D raster image using our polygon-based Triangle-Mesh-Rasterization-Projection (TMRP) method [39]. The result is the ground truth disparity map.

**Figure 10:**
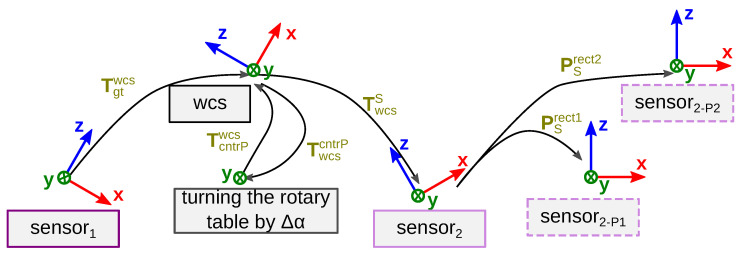
Homogeneous coordinate transformations $T_{\mathrm{from}}^{\mathrm{to}}$ to generate ground truth disparities for *TranSpec3D* data set. Sensor_1_$\hat{=}$ thermal 3D sensor; gt $\hat{=}$ ground truth depth (sensor_1_); wcs $\hat{=}$ world coordinate system; cntrP $\hat{=}$ central point of the turning axis of the rotary table; sensor_2_$\hat{=}$ NIR 3D sensor; S $\hat{=}$ sensor_2_; $P_{S}^{\mathrm{rect}1/2}$$\hat{=}$ projection matrices of sensor_2_.

In the following, we write vectors in bold lower-case (o) and matrices using bold-face capitals (O). Three-dimensional rigid body transformations that bring points from the coordinate system *a* into the coordinate system *b* are denoted by $T_{a}^{b}$, where T stands for “transformation” (according to [40]). Figure 10 shows an overview of the homogeneous coordinated transformations details.

##### Homogeneous Coordinate Transformation

Equation (7) describes the matrix $R_{\mathrm{axisY}}$ consisting of components of the rotation vector $r_{axisY}$, and Equations (2) and (8) describe the rotation matrix $R_{y(\Delta \alpha )}$ using the Rodrigues’ rotation formula [41], where I is the unit matrix:

(7) $$R_{\mathrm{axisY}}=\left[\begin{array}{ccc} 0 & -r_{z} & r_{y}\\ r_{z} & 0 & -r_{x}\\ -r_{y} & r_{x} & 0 \end{array}\right]$$

(8) $$R_{y(\Delta \alpha )}=I+\sin\left(\theta \right)\;\cdot \;R_{\mathrm{axisY}}+\left(1-\cos\left(\theta \right)\right)\;\cdot \;R_{\mathrm{axisY}}^{2}\;\;\mathrm{with}\;\theta =-\Delta \alpha \;\cdot \;\frac{\pi }{180}$$

Equation (11) describes the homogeneous coordinates transformation $T_{{\mathrm{sensor}}_{1}}^{{\mathrm{sensor}}_{2}}$ of the ground truth point $P_{\mathrm{gt}}$ mathematically. The required transformation matrices are described in Equations (9) and (10):

(9) $$T_{\mathrm{gt}}^{\mathrm{wcs}}=\left[\begin{array}{cc} {\left(R_{\mathrm{gt}}^{\mathrm{wcs}}\right)}^{-1} & -{\left(R_{\mathrm{gt}}^{\mathrm{wcs}}\right)}^{-1}\;\cdot \;t_{\mathrm{gt}}^{\mathrm{wcs}}\\ 0_{1\times 3} & 1 \end{array}\right],\;T_{S}^{\mathrm{wcs}}=\left[\begin{array}{cc} R_{\mathrm{wcs}}^{S} & t_{S}^{\mathrm{wcs}}\\ 0_{1\times 3} & 1 \end{array}\right]$$

(10) $$T_{\mathrm{wcs}}^{\mathrm{cntrP}}=\underset{\mathrm{rotation}\mathrm{at}\Delta \alpha \mathrm{in}\mathrm{at}y\mathrm{axis}}{\underset{︸}{\left[\begin{array}{cc} R_{y(}\Delta \alpha ) & t_{\mathrm{cntrP}}\\ 0_{1\times 3} & 1 \end{array}\right]}},\;T_{\mathrm{cntrP}}^{\mathrm{wcs}}=\underset{\mathrm{retransformation}\mathrm{to}\mathrm{origin}}{\underset{︸}{\left[\begin{array}{cc} I & -t_{\mathrm{cntrP}}\\ 0_{1\times 3} & 1 \end{array}\right]}}$$

(11) $$T_{{\mathrm{sensor}}_{1}}^{{\mathrm{sensor}}_{2}}=T_{\mathrm{gt}}^{S}=T_{\mathrm{wcs}}^{S}\;\cdot \;\underset{\mathrm{turning}\mathrm{by}\Delta \alpha }{\underset{︸}{T_{\mathrm{cntrP}}^{\mathrm{wcs}}\;\cdot \;T_{\mathrm{wcs}}^{\mathrm{cntrP}}}}\;\cdot \;T_{\mathrm{gt}}^{\mathrm{wcs}}\;,\;\;\left(\begin{array}{c} X_{\mathrm{gt}2S}\\ Y_{\mathrm{gt}2S}\\ Z_{\mathrm{gt}2S}\\ 1 \end{array}\right)=T_{\mathrm{gt}}^{S}\;\cdot \;\underset{\mathrm{point}P_{\mathrm{gt}}}{\underset{︸}{\left(\begin{array}{c} X_{\mathrm{gt}}\\ Y_{\mathrm{gt}}\\ Z_{\mathrm{gt}}\\ 1 \end{array}\right)}}$$

##### Conversion Depth to Disparity

This step only applies if sensor_2_ is a stereo system and not a monocular 3D sensor, such as an RGB-D sensor, since a stereo data set will require disparity maps as ground truth. Equation (12) describes the homogeneous transformation and projection applied to the 3D point $P_{\mathrm{gt}}$ of the sensor_1_. Thus, the point is projected from the rectified first camera coordinate system of the sensor_1_ into the first and second rectified camera images of the sensor_2_. $P_{S}^{\mathrm{rect}1}$ and $P_{S}^{\mathrm{rect}2}$ are the 3×4 projection matrices (see description of openCV *stereoRectify()* function). We created these two projection matrices of the reprojection matrix Q (Section 3.2.1):

(12) $$\left(\begin{array}{c} X_{\mathrm{Pl}}\\ Y_{\mathrm{Pl}}\\ Z_{\mathrm{Pl}} \end{array}\right)=\underset{\mathrm{projection}\;\mathrm{to}\;\mathrm{left}}{\underset{︸}{P_{S}^{\mathrm{rect}1}}}\;\cdot \;T_{\mathrm{gt}}^{S}\;\cdot \;\left(\begin{array}{c} X_{\mathrm{gt}}\\ Y_{\mathrm{gt}}\\ Z_{\mathrm{gt}}\\ 1 \end{array}\right),\;\left(\begin{array}{c} X_{\mathrm{Pr}}\\ Y_{\mathrm{Pr}}\\ Z_{\mathrm{Pr}} \end{array}\right)=\underset{\mathrm{projection}\;\mathrm{to}\;\mathrm{right}}{\underset{︸}{P_{S}^{\mathrm{rect}2}}}\;\cdot \;T_{\mathrm{gt}}^{S}\;\cdot \;\left(\begin{array}{c} X_{\mathrm{gt}}\\ Y_{\mathrm{gt}}\\ Z_{\mathrm{gt}}\\ 1 \end{array}\right)$$

(13) $$\left(\begin{array}{c} x_{\mathrm{Pl}}\\ y_{\mathrm{Pl}} \end{array}\right)=\left(\begin{array}{c} X_{\mathrm{Pl}}/Z_{\mathrm{Pl}}\\ Y_{\mathrm{Pl}}/Z_{\mathrm{Pl}} \end{array}\right),\;\;\left(\begin{array}{c} x_{\mathrm{Pr}}\\ y_{\mathrm{Pr}} \end{array}\right)=\left(\begin{array}{c} X_{\mathrm{Pr}}/Z_{\mathrm{Pr}}\\ Y_{\mathrm{Pr}}/Z_{\mathrm{Pr}} \end{array}\right)$$

Equation (14) describes the 2D point cloud with the ground truth disparity values $d_{x}$. $rx_{\mathrm{gt}}$ and $ry_{\mathrm{gt}}$ are the 2D coordinates of the raw points $P_{\mathrm{gt}}$ of sensor_1_ (see Equation (11):

(14) $$\left(\begin{array}{c} x_{\mathrm{Pl}}\\ y_{\mathrm{Pl}}\\ d_{x}\\ rx_{\mathrm{gt}}\\ ry_{\mathrm{gt}} \end{array}\right)\overset{\mathrm{pseudo}\text{-}\mathrm{real}\;\mathrm{ground}\;\mathrm{truth}}{\leftarrow }\;\underset{\mathrm{calculate}\;\mathrm{pseudo}-\mathrm{real}\;\mathrm{disparity}\;d_{x}\;\mathrm{based}\;\mathrm{on}\;\mathrm{two}\;\mathrm{point}\;\mathrm{clouds}\;({\mathrm{points}}_{\mathrm{Pl}}\;\mathrm{and}\;{\mathrm{points}}_{\mathrm{Pr}})}{\underset{︸}{d_{x}\left(x_{\mathrm{Pl}},y_{\mathrm{Pl}}\right)=\left\{\begin{array}{cc} x_{\mathrm{Pl}}\left(y_{\mathrm{Pl}}\right)-x_{\mathrm{Pr}}\left(y_{\mathrm{Pl}}\right) & \left(d_{x}\geq 0\right)\\ \mathrm{NaN} & \left(d_{x}<0\right) \end{array}\right.,\;\mathrm{for}\;\left(y_{\mathrm{Pl}}==y_{\mathrm{Pr}}\right)}}$$

##### Projection of Plane Cloud into 2D Raster Image

The low-resolution ( 0.3 Mpx) transformed point cloud $P(x,y,d_{x},rx,ry)$ is projected into a dense, accurate 2D disparity map with an image resolution of 1680 px × 1304 px. We use our polygon-based Triangle-Mesh-Rasterization-Projection (TMRP) method [39,42]. The generated disparity map is created with eight decimal places. The disparity map is saved as an M-alpha image. The alpha channel indicates the valid gaps. Disparity maps are saved as PNG and PFM files.

## 4. TranSpec3D Data Set

### 4.1. Data Set Statistics

**Figure 11:**
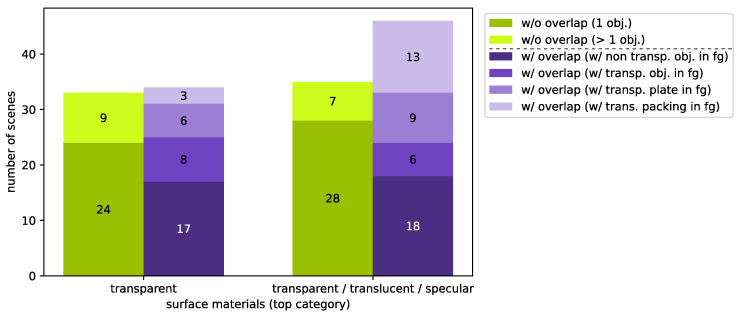
Classification of the captured scenes according to the surface material (top category) and complexity of the scene (lower category). A scene consists of six to seven pairs of images with different lighting. Transparent (transp.); object (obj.); foreground (fg).

**Figure A3:**
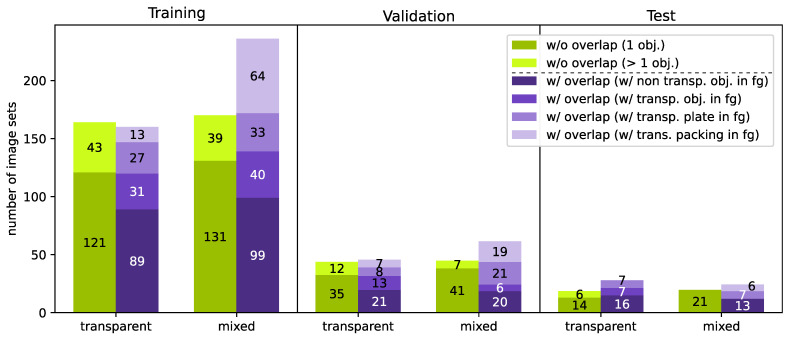
Training data set (730 image sets), validation data set (210 image sets) and test data set (97 image sets). A image set consists of an image pair and a disparity map (ground truth).

**Figure A4:**
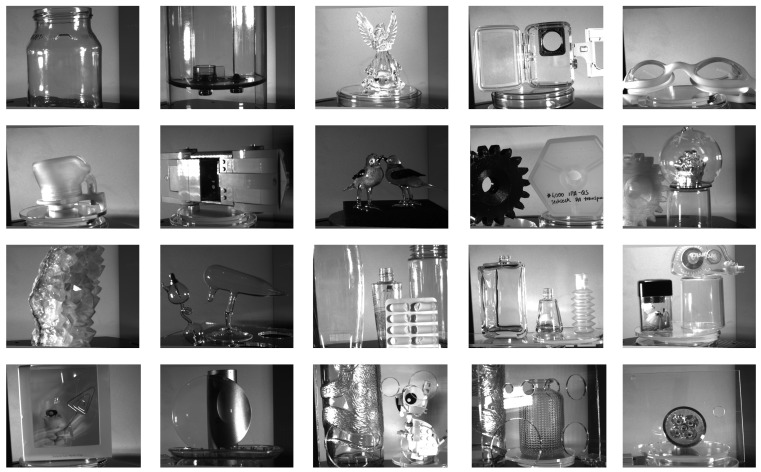
Examples of collected images of *TranSpec3D* data set (only undistorted and rectified NIR images).

Our real stereo data set *TranSpec3D* contains the raw as well as the undistorted and rectified stereo images (NIR 3D sensor) and the ground truth disparity maps. Our data set includes 110 objects with transparent or specular surfaces. We have various glass objects (vases, drinking glasses, chemical utensils, and historical glass objects), various transparent and translucent plastics (packaging boxes or technical objects), medical objects (clear orthodontic aligners), mirror objects, etc. Figure A4 shows examples of captured objects and scenes from our data set. The split of the data: 70% as the training data set, 20% as the validation data set and 10% as the test data set. For a balanced split, we categorized the captured scenes by surface materials (top category) and complexity (sub category). On average, a scene consists of six to seven pairs of images with different lighting, which means that the stereo images have different reflections. The top category distinguishes between transparent (exclusively or strongly predominant) and mixed surface materials (transparent, translucent and reflective). The subcategory defines the complexity of the scene. Here, it is roughly divided into “without” and “with overlap”. A further subdivision is made in the subcategory “without overlap” between one object and several objects and in the subcategory “with overlap” between the complexity of the objects in the foreground. Figure 11 shows the subdivision into the top and subcategories. The validation and test data set contains data from each category according to the percentage. We made sure that all three data sets contain unique objects (novel objects unseen in training). Figure A3 (Appendix E) shows the distribution of the training/validation/test according to the categories.

### 4.2. Accuracy Assessment

The characterization of the thermal 3D sensor (sensors_1_) is carried out according to the VDI/VDE guideline 2634. For a field size of 160 mm  × 128 mm (horizontal × vertical) the measurement quality is 10 μm to 150 μm [43]. The characterization of the NIR 3D sensor (sensors_2_) is determined using the test specimen (b) from Figure 6. We calculate the 3D point standard deviation of the measured plane front surface to an ideal plane. The 3D standard deviation is about 42 μm.

### 4.3. Comparison of TranSpec3d Data Set with Booster Data Set

To show the advantages and disadvantages of our measurement principle and data set *TranSpec3D* compared to the *Booster* data set [18], we compared different properties and present them in Table 4. Compared to the generation of the *Booster* data set, our measurement principle has significant advantages in terms of time and effort for the generation of the ground truth data. By eliminating the object preparation, steps three and four are skipped in the acquisition pipeline. This also eliminates the influence of error due to possible inaccurate positioning of the prepared object (step four) at the original position of the acquired transparent object (step two). In addition, our method does not damage the objects, which is resource conserving and especially important for sensitive objects, such as historical glass. However, a disadvantage can be the lower resolution of the thermal 3D sensor compared to the NIR stereo system. Therefore, the low-resolution raw depth of the thermal 3D sensor must be extrapolated to the high resolution of the NIR 3D sensor. To achieve high accuracy and density, we use the polygon-based TMRP method (including up-sampling) [39]. This possible error influence is not present in the *Booster* data set since, here, the ground truth data are generated due to the object preparation with classic active stereo (based on six RGB projectors). Another limitation of our measuring principle is the measurement volume. This is limited by the safety-related enclosure of the thermal 3D sensor (Section 5.2.1). Therefore, our data set contains only laboratory scenes.

## 5. Discussion

### 5.1. Advantages of Our New Proposed Measuring Principle TranSpec3D

We created a real stereo data set *TranSpec3D* for transparent and specular objects using ground truth without object preparation. To our knowledge, our novel measurement principle *TranSpec3D* is the **first-ever** method to generate real (non-synthetic) monocular or stereo data sets with ground truth values (depth or disparity maps) for transparent and specular objects. To achieve this, we used an additional thermal 3D sensor (sensor_1_) developed by Landmann et al. [30] to a conventional 3D sensor (sensor_2_). The advantages of this sensor are as follows:

- **No preparation of free-form surfaces necessary for the first time;**
- Heating locally and only a few Kelvin;
- Fully automatic measurement in seconds with subsequent automated evaluation;
- Objects from different materials measurable;
- High accuracy;
- Still a current research topic with high potential for improvement.

The presented new measurement principle *TranSpec3D* can be used to create stereo data sets with ground truth disparity for *stereo matching* [21,34] as well as monocular data sets with ground truth depth for *depth-from-mono* [22,23,24]. Depending on the application, only the conventional 3D sensor (Figure 4, sensor_2_) has to be replaced or extended. For a monocular data set, for example, an RGB-D sensor can be used (cf. [12,32]). For a stereo data set, for example, an RGB stereo system with a parallel or convergent camera arrangement can be used (cf. [18]).

### 5.2. Limitations

Creating a data set for transparent and specular objects with our novel measurement principle does not require object preparation, which saves object resources and a lot of time (see Section 2.1). In addition, the surface of the object is not prepared by, for example, the varnish. But there are limitations. Section 5.2.1 describes the limitation of the method and our data set due to the thermal 3D sensor. Section 5.2.2 describes the limitation of our data set due to the current measurement setup.

#### 5.2.1. Due to the Thermal 3D Sensor

- Physical limitations of thermal 3D sensor technology: The measured accuracy of the measured depth values depend mainly on two material parameters: complex refractive index *n* (a material and wavelength-dependent (dispersion) quantity, which is composed of the *real refractive index*$n^{\prime }$ and the *absorption index*κ as follows $n=n^{\prime }+i\kappa$) and thermal conductivity. The objects must not be transmissive at the irradiation wavelength (10.6 μm, see Table A2), and must reflect a sufficiently large portion. In addition, the object must be heatable close to the surface, and heat should not disappear immediately. This means objects with very high thermal conductivity, e.g., metals or ceramics, cannot be captured accurately [31].
- Measurement volume limitations: Due to the CO_2_ laser (40 W), the measuring system must be enclosed for safety reasons. Therefore, this method is limited to objects of a certain size and to laboratory scenes (indoor/outdoor).
- Low resolution of the 3D point cloud: The resolution of the raw depth point cloud ( 0.3 Mpx), which is the basis for the ground truth disparity of our data set, is very low due to the thermal imaging cameras (FLIR A6753sc). When using a VIS/NIR 3D sensor with a much higher resolution than 0.3 Mpx, we lose information and therefore accuracy due to under-sampling despite the application of the TMRP algorithm [39].
- High hardware costs: The thermal 3D sensor is very expensive. The high cost share is due to the cooled thermal cameras (FLIR A6753sc) and will probably decrease in the next few years due to further developments. In the future, more affordable technologies will make it possible to create customized training data sets, e.g., for small series, directly on site.

#### 5.2.2. Due to the Current Measurement Setup

The following limitation refer to our current measurement setup (Figure 4). However, the current limitations will be resolved in the future. Measuring volume and environment: Our current measurement setup can only measure static objects under laboratory conditions (indoors and outdoors) with a measurement volume of 160 mm×128 mm×100 mm. Currently, the mid-wave infrared (MWIR) cameras (sensor_1_) at 125 fps are the limiting component to also capture dynamic objects. The use of high-speed LWIR cameras at 1000 fps handles this limitation and shows a dynamic measurement process of a crushing bottle with a 20 fps 3D rate [46].

### 5.3. Open Question

There are two approaches to creating the ground truth of a real monocular or stereo data set (see Table A1, Appendix B). These are shown in Figure 1. Which approach is better in terms of ground truth data accuracy: (a) the state-of-the-art approach with manipulation of the surface by object preparation as with the booster data set [18], or (b) our approach based on the thermal 3D sensor [30] (cf. Figure 1)?

### 5.4. Future Work

- In the future, we want to quantitatively investigate the difference in the 3D point clouds of objects with and without object preparation (cf. Section 5.3).
- In the future, we want to expand our measurement system as follows:
  – With further modalities, such as an RGB stereo system in the VIS range and a polarization camera for segmenting the transparent objects [47,48].
  – With different backgrounds to obtain a data set with different appearances of glass objects [6,49]. To generate disparity values from the background, in the future, the disparity map of sensor_2_ should be merged with the current one to have 100% “visual” density (merge similar to [12]).
  – With a rotary table without a spindle.
- With our new measurement principle, additional real data sets can also be created for further optical uncooperative objects in the VIS or NIR range, e.g., black objects.

## 6. Conclusions

In various applications, such as human–robot interactions, autonomous robot navigation or autonomous waste recycling, the perception or 3D reconstruction of transparent and specular objects is required. However, such objects are optically uncooperative in the VIS and NIR ranges. The capture of transparent surfaces is still a corner case in stereo matching [18,19]. This can also be seen by the fact that most deep stereo matching networks perform worse on transparent and other visually uncooperative objects [20,29]. This is also due to the fact that the generation of real data sets with ground truth disparity maps is very time consuming and costly due to a necessary object preparation (or an additional opaque twin), which is reflected in the small number of available data sets (Table 1). For this reason, we introduce our novel measurement principle *TranSpec3D* that **accelerates and simplifies the generation of a stereo or monocular data set with real measured ground truth** for transparent and specular objects without the state-of-the-art object preparation [18,25] or an additional opaque twin [2,14]. In contrast to conventional techniques that require object preparation [18,25], opaque twins [2,14] or 3D models [12,32] to generate a ground truth, we obtain the ground truth using an additional thermal 3D sensor developed by [30]. The thermal 3D sensor captures the optically uncooperative objects three-dimensionally without time-consuming object preparation. With our measurement principle, the time and effort required to create the data set is massively reduced. The time-consuming object preparation as well as the time-consuming object placement of transparent and opaque objects is eliminated. In addition, the surface of the object is not manipulated, which means that sensitive objects can also be detected, e.g., historical glass. Another special feature is the generalizability of the measurement principle, i.e., any conventional 3D sensor in VIS or NIR (sensor_2_) can be extended with the thermal 3D sensor (sensor_1_), e.g., by a RGB stereo system (with parallel or convergent camera arrangement) for a stereo data set with ground truth disparity values (for deep stereo matching [21]) or by a RGB-D sensor for monocular data sets with ground truth depth values (for monocular depth estimation [4,23,24]). In addition, there is a high development potential to optimize the thermal 3D sensor to make this technology as accessible as possible.

We apply this measurement principle to generate our data set *TranSpec3D*. For this, we use a conventional NIR 3D sensor and the thermal 3D sensor [30]. To enlarge the data set naturally (data augmentation), we record each scene with different NIR emitter positions. After the data collection, a data analysis and annotation of ground truth disparities takes place. To ensure that the ground truth disparity map has the same resolution as the stereo images, the Triangle-Mesh-Rasterization-Projection (TMRP) method [39] is used. Our data set *TranSpec3D* consists of stereo imagery (raw as well as undistorted and rectified) and ground truth disparity maps. We capture 110 different objects (transparent, translucent or specular) at different illumination environments, thus increasing the data set and creating different reflections on the surface (natural data augmentation). We categorize the captured 148 scenes by surface materials (top category) and complexity (sub category) (Figure 11). This allows us to have a balanced split of the data set into training/validation/test data sets (Figure A3). Our data set consists of 1037 image sets (consisting of stereo image and disparity map). Our data set is available at https://QBV-tu-ilmenau.github.io/TranSpec3D-web (accessed on 6 September 2023). We present the advantages and disadvantages of our method by comparing our *TranSpec3D* data set with the *Booster* data set [18] (cf. Table 4).

## Figures and Tables

**Table 1:** Overview of existing real stereo data sets for transparent objects and for disparity estimation (with ground truth). Transparent Object Data Set (TOD) [25]—first method of keypoint-based pose estimation for transparent 3D objects from stereo RGB images. *Booster* data set exclusively for disparity estimation [18]. *TranSpec3D* data set with ground truth first-ever generated without object preparation (obj. prep.). All data sets show indoor scenes. # Objects refers to the number of objects. Material: transparent objects (T); translucent objects (Tl); specular objects (S).

Data Set	# Objects	Type of Material	Scene Type	Ground Truth	Stereo Modality	Camera Arrangement
TOD [25]	15	(T)	single obj.	opaque twin ${}^{†}$	RGB	parallel
Booster [17,18]	606	(T) + (Tl) + (S)	scene	w/ obj. prep.	RGB	parallel
TranSpec3D (ours)	110	(T) + (Tl) + (S)	1–5 obj., partly overlap	**w/o obj. prep.** ${}^{‡}$	NIR	convergent

${}^{†}$ only depth maps of transparent and opaque object (RGB-D using a Microsoft Azure Kinect sensor) instead of disparities; ${}^{‡}$ with an additional thermal 3D sensor.

**Table 2:** Overview of existing real-world mono data sets for transparent objects with ground truth depth. *ClearGrasp-Real* data set [2] for robot manupulation (grasping tasks). *Toronto transparent objects depth data set* (TODD) [12], a large-scale real Transparent Object Data Set. *TRANS-AFF*, an affordance data set for transparent objects [14]. *STD data set* [32], composed of transparent, specular, and diffuse objects. All data sets show indoor scenes. # Objects and # Samples refers to the number of objects and samples. (S), (T) and (D) refer to specular, transparent and diffuse materials.

Data Set	# Objects / # Samples	# Type of Material	Scene Type	Ground Truth	Modality RGB-D (RealSens)
ClearGrasp-Real [2]	10/286	(T) + (D)	1–6 objects	opaque twin ${}^{†}$	D415
TODD [12]	6/ 1.5 k	(T)	1–3 obj., overlap	3D model ${}^{‡}$	D415
TRANS-AFF [14]	NN/ 1.3 k	(T)	single obj.	opaque twin	D435i & D415
STD [32]	50/ 27 k	(S) + (T) + (D)	>4 obj., cluttered	3D model ${}^{§}$	D415

${}^{†}$ spray-painting; replacing of opaque object with a GUI app, see [2]; ${}^{‡}$ due to AprilTags on the base template, the 3D model of the object(s) can be adjusted to the appropriate locations to complete the ground truth depth; ${}^{§}$ due object capture API on macOS [32].

**Table 3:** Overview of calibration methods used.

	Thermal 3D Sensor	NIR 3D Sensor
	**(sensor_1_)**	**(sensor_2_)**
method/process	bundle adjustment (cond.: only 1 static pattern over time)	according to Zhang [38]
target pattern (Figure A2)	ArUco marker and symmetrical circles	checkerboard with three circle marker
target material	circuit board FR-4 w/ copper	glass plate

**Table 4:** Comparison of two available real stereo data sets for transparent and specular surfaces.

	Booster [44]	TranSpec3D (Ours)
	**(w/ Object Preparation)**	**(w/o Object Preparation)**
**Data set acquisition pipeline**		
1. Calibration	**only one 3D sensor**	two 3D sensors
2. Acquisition w/o obj. prep.	only stereo images	stereo images **and raw depth**
3. Object/scene preparation	necessary ${}^{†}$	**-**
4. Acquisition w/ obj. prep.	for gt (obj. positioning ${}^{†}$)	**-**
5. Data analysis & annotation	**normal process**	+ convert raw depth to gt
Required time	very high	**low**
Required effort	very high	**low**
**Object**		
Preparation	required	**not required**
Reusability	no → mostly irreparable	**yes**
w/high thermal conductivity	**detectable**	not detectable (Section 5.2.1)
w/low optical density	**detectable**	not detectable
**Ground truth**		
Influence on ground truth	manipulated surface	real surface (Section 5.2.1)
Density of ground truth image	**dense**	**dense**, but w/o background
Resolution of raw depth	**same to images**	lower ( 0.37 MPx)
Resolution of ground truth	**same to images**	**same to images** (but w/up-sampling [39])
**Experimental setup**		
Additional hardware	6x projectors; spray	thermal 3D sensor [45]
Measuring volume (indoor)	**not limited**	limited (only laboratory ‡)
Measuring volume (outdoor)	-	**limited (only laboratory ‡)**
Camera arrangement	parallel	convergent
Wavelength of stereo system	VIS	NIR, λ=850 nm
Image resolution	12.4 Mpx/ 2.3 Mpx	2.2 Mpx
Baseline of stereo system	80 mm/ 40 mm	130 mm
**Costs**	high personnel costs and obj. consumption	very high hardware costs (Section 5.2.1)

${}^{†}$ time intensive; ‡ technology also works in sunlight; “laboratory condition” only refers to the scenery of the objects, which is given by the safety-related enclosure (CO_2_ laser of the thermal 3D sensor).

## Data Availability

Our data set *TranSpec3D* is available at https://QBV-tu-ilmenau.github.io/TranSpec3D-web (accessed on 6 September 2023).

## Supplementary Materials

The following supporting information can be downloaded at: https://www.mdpi.com/article/10.3390/s23208567/s1, Figure S1. “The strange effects of resolution” according to Miangoleh et al. [50].

## Abbreviations

The following abbreviations are used in this manuscript:

A0/A1/A2	approaches 0/1/2
AI	artificial intelligence
CO_2_	carbon dioxide
coord.	coordinate
(D)	diffuse objects
2D/3D	two/three dimensional
FR	flame retardant
fg	foreground
GOBO	goes before optics
gt	ground truth
img.	image
MWIR	mid-wave infrared
n_*_	refractive index of *
NaN	not a number
NIR	near infrared
NN	non nominatus
obj.	object
PFM	Portable FloatMap
PMMA	polymethyl methacrylate
prep.	preparation
proj.	projection
(R)	reflective objects
rep.	representatives
res.	resolution
RGB-D	image with four channels: red, blue, green, depth
ROI	region of interest
SOTA	state of the art
suppl.	supplementary
(T)	transparent objects
(Tl)	translucent objects
TMRP	Triangle-Mesh-Rasterization-Projection
transp.	transparent
TOD	Transparent Object Data Set [12]
VIS	visible spectrum
wcs	world coordinate system

## Appendix A. Limitations of Active 3D Sensors in VIS and NIR with Optically Uncooperative Objects

Figure A1 shows the limitations of active 3D sensors in VIS or NIR with optically uncooperative objects. Because the projection patterns are not visible, no correspondence points can be found (see *depth error I* in Section 2.1).

## Appendix B. Approaches to 3D Detection of Optically Uncooperative Objects

Table A1 compares three approaches for the 3D detection of optically uncooperative objects in the VIS and NIR spectral ranges.

A0: This approach uses conventional stereo systems in the VIS and NIR and prepared objects; see Figure 1a.
A1: This approach continues to use conventional stereo systems, but due to the deep stereo matching approaches, no object preparation is necessary during the process.
A2: This approach captures transparent objects with alternative measurement technologies [30]. No object preparation and no data set are necessary; see Figure 1b.

**Table A1:** Approaches to minimize resp. solve the current limitation of stereo vision, the optically uncooperative surfaces in the visible spectral range.

	Approach A0	Approach A1	Approach A2
Systems	conventional	conventional	thermal 3D sensor [30]
Stereo matching	model based	data driven (AI based)	s. [30]
Object preparation	necessary	yes (A0)/no (A2)	no
Application: For in-line quality control or dynamic processes	(no)	**possible**	too long measurement time
Application area (in general)	**flexible**	**flexible**	limited (required housing)
Task type	-	**outdoor/indoor**	indoor, laboratory
Flexibility of transparent objects	-	lower, depending on data set and training	**high**, limited due to technology †
Transparent objects measurable	w/preparation	only w/AI	**measurable w/o preparation** †
data set (training and testing)	no	required ${}^{‡}$	**not required**
Objects of the data set: still usable	no	no ${}^{§}$/**yes** (TranSpec3D)	**yes** *
Raw res. of ground truth vs. image	**-**	**same**	lower (TMRP [39])
Hardware costs	low	**middle**	very high
Representatives	-	Booster [18]; our TranSpec3D	thermal 3D sensor [30]

† limitations when using the thermal 3D sensor, see Section 5.2.1; ${}^{‡}$ (i) synthetic or (ii) real-world data set; (ii) currently very rare (Booster data set [18]) or cost- and time intensive for own generation; ${}^{§}$ only for training: object painting/powdering [17,18]; * Object is not destroyed by heat input [51].

## Appendix C. Sensor Specifications

Table A2 shows the properties of our sensors utilized to generate the *TranSpec3D* data set (see measurement setup, Figure 4).

**Table A2:** Properties of used measuring systems.

Properties	Thermal 3D Sensor [45]	NIR 3D Sensor [36]	Monocular Camera (Optional)
	**sensor_1_**	**sensor_2_**	**camera_3_**
system	FLIR A6753sc	Blackfly®S USB3 ${}^{†}$	
stereo vision arrangement	convergent	convergent	-
base distance	211 mm	130 mm	-
image size (raw)	640 px × 512 px	1616 px × 1240 px	1616 px × 1240 px
image size (rectified)	696 px × 534 px	1680 px × 1304 px	-
λ of image acquisition	MWIR, 3 μm–5 μm	850 nm	400 nm–700 nm
λ of projector	10.6 μ m	850 nm	-
pattern projection	sequential fringe projection [30]	GOBO	-

${}^{†}$ Modell: BFS-U3-20S4M-C: 2.0 MP, 175 FPS, Sony IMX422, Mono, https://www.flir.de/products/blackfly-s-usb3/?model=BFS-U3-20S4M-C&vertical=machine+vision&segment=iis (accessed on 1 September 2023).

## Appendix D. Sensor Calibration and Test Specimens

Figure A2 shows the utilized calibration targets. Table A3 shows the details of the test specimen spherer bar.

**Table A3:** Details of the test specimen ball bar or sphere bar (Figure A2).

Spherical Bar	$l_{\mathrm{distance}-\mathrm{of}-\mathrm{ball}-\mathrm{cent}.}$	$d_{1}$	$d_{2}$
set value	100.06908 m m	14.99727 m m	14.99929 m m

**Table A4:** Details of the test specimen for determining the world coordinated system (wcs) of *TranSpec3D* data set.

Four Hemispheres Specimen	*d*	$l_{\mathrm{point}-\mathrm{to}-\mathrm{point}}$
set value	40 mm	60.1 m m
actual value	σ=20 μm to 30 μm	60.2 m m

## Appendix E. TranSpec3D Data Set

Figure A3 shows the distribution of the *TranSpec3D* data set (70.4% training/20.2% validation/9.4% test). Figure A4 shows examples of the captured objects and scenes (NIR images).
