# Supplementary material for: TranSpec3D: A Novel Measurement Principle to Generate A Non-Synthetic Data Set of Transparent and Specular Surfaces without Object Preparation
Source: Sensors (Basel). 2023 Oct 18;23(20):8567. doi: 10.3390/s23208567 (PMC10611300; doi:10.3390/s23208567)
Supplement: Supplementary file 1 [file sensors-23-08567-s001.zip › sensors-2627246-supplementary.pdf]

Supplementary Materials: TranSpec3D: A novel measurement principle to generate a non-synthetic data set of transparent and specular surfaces without object preparation

Christina Junger [1,*], Henri Speck[2], Martin Landmann[2], Kevin Srokos[2] and Gunther Notni [1,2,*]

1. Strange Effects of Resolution

Figure S1 visualizes "The Strange Effects of Resolution".

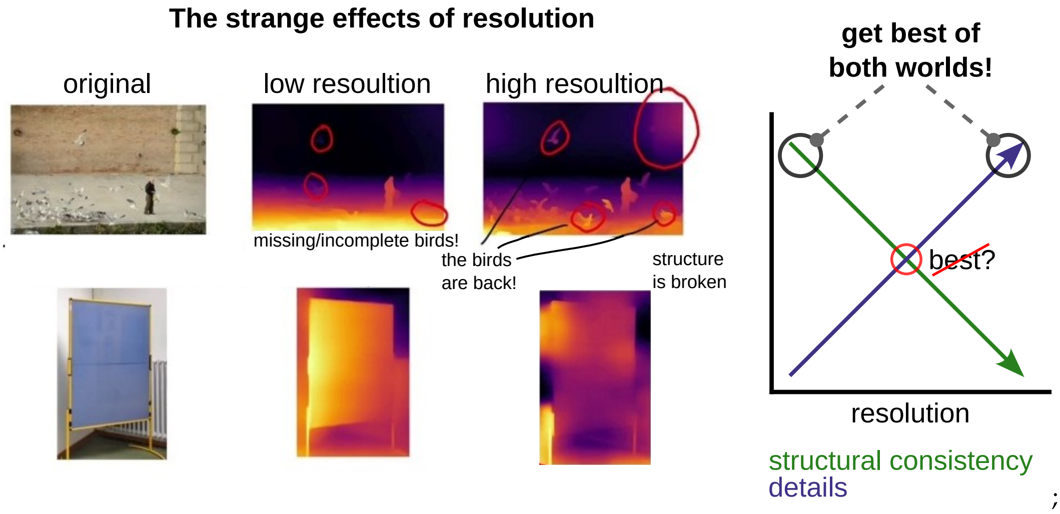

Figure S1. "The strange effects of resolution" according to Miangoleh et al. http://yaksoy.github.io/highresdepth/CVPR21PosterSm.jpg.
